# An introduction to systematic reviews and meta-analysis: A workshop report on promoting evidence based medical practice through capacity building in research synthesis

**DOI:** 10.4314/pamj.v8i1.71066

**Published:** 2011-02-24

**Authors:** Lawrence Mbuagbaw, Charles Shey Wiysonge, Dickson Shey Nsagha, Pierre Ongolo-Zogo, Tomas Pantoja

**Affiliations:** 1 Centre for the Development of Best Practices in Health, Yaoundé Central Hospital, PO Box 87, Henri Dunant Avenue, Messa, Yaoundé, Cameroon; 2 School of Child and Adolescent Health and Institute of Infectious Disease and Molecular Medicine, University of Cape Town, South Africa; 3 Department of Public Health and Hygiene, Faculty of Health Sciences, University of Buea, PO Box 63, Buea, Cameroon; 4 Department of Family Medicine, Pontifica Universidad Catolica de Chile

**Keywords:** Systematic reviews, meta-analysis, workshop, capacity building, Cameroon

## Abstract

The increasing urgency for evidence based practice, especially in resource limited settings has inspired many initiatives to this effect. In Africa there is limited skill in research synthesis and the production of systematic reviews. The Centre for the Development of Best Practices in Health, together with the South African Cochrane Centre organised a workshop to train Cameroonian researchers on how to initiate and complete systematic reviews. Five facilitators and fifteen participants met over a period of four days. At the end of the workshop the participants expressed high levels of satisfaction and motivation to conduct systematic reviews, but expressed the need for additional support. Facilitators of future systematic review courses should address challenges related to internet access, adult education and realistic expectations from the participants.

## Introduction

Over the past few decades, the need for reliable information to guide health care decision making has spawned the art of research synthesis. Even though the idea and practice of research synthesis are not new [1], the systematization of the process is fairly recent. The most outstanding product of research synthesis is the systematic review. A systematic review is “a review in which bias has been reduced by the systematic identification, appraisal, synthesis, and, if relevant statistical aggregation of all relevant studies on a specific topic according to a predetermined and explicit method [2].” This would seem to be an invaluable resource for developing countries. They would spend their limited resources only on the most effective interventions and policies [3]. Unfortunately, the expertise for conducting systematic reviews is limited in Africa [4]. Scientists in less wealthy countries are not familiar with reviews, internet access is limited and costly, library services are poorly resourced and there is limited training and support [5]. The South African Cochrane Centre (SACC) has taken the lead in providing training opportunities and support for potential and current authors of systematic reviews from Africa [4]. The SACC and the Centre for the Development of Best Practices in Health (CDBPH) jointly organized a systematic review training workshop in January 2011 for University lecturers and researchers in medical schools and other health institutions in Cameroon. The CDBPH is a research unit based at the Yaoundé Central Hospital in Cameroon which was created, with funding from a Global Health Leadership Award from the International Development Research Centre (IDRC) – Canada, to promote evidence-informed health decision making in Cameroon. The CDBPH is therefore a knowledge translation and exchange centre aimed at facilitating interaction between health researchers and decision-makers. This initiative offers researchers the opportunity to collect, synthesize and disseminate research evidence in user-friendly formats.

## Workshop report

**Aim of the workshop**

The aim of the workshop was to build systematic review skills in Cameroon, by providing participants with the ability to: 1) distinguish between a traditional review and a systematic review, 2) understand the methodology involved in conducting a systematic review, 3) access systematic reviews, 4) critically appraise a systematic review, 5) interpret the results of a systematic review, 6) consider conducting a systematic review, 7) identify a priority topic for a systematic review, and, 8) register a title for a systematic review.

**Participants**

The CDBPH sent out workshop invitations to the deans of the medical schools of the universities of Buea, Douala and Yaoundé I as well as potential systematic review authors in the Ministry of Public Health. Fifteen university lecturers and researchers attended the workshop. A majority of the participants (12/15) were clinicians.

**Facilitators**

The facilitators were chosen for their content and methods expertise and came from diverse locations. CSW is a Cochrane Review author and researcher at the University of Cape Town in South Africa, DN is a Cochrane Review author and lecturer at the University of Buea in Cameroon, TP is a Cochrane Review author and researcher from the “Pontifica Universidad Catolica” in Chile and both LM and POZ are Cochrane Review authors and researchers from the CDBPH.

**Pre-workshop tasks**

Three weeks before the workshop, participants were asked to identify possible topics for systematic reviews in clinical medicine or health systems research.

**Program**

This four-day training workshop provided participants with a basis in the design, analysis, and interpretation of systematic reviews of health research. Participation was interactive and presentations were done in both English and French. Participants were given grounding in all aspects involved in conducting a systematic review and meta-analysis, and had the opportunity to gain practical experience of the tasks involved. On the fourth day, more emphasis was laid on reviews of health systems and organisation of care. By the end of the workshop, participants were equipped with the necessary skills to conduct their own high quality systematic reviews of health research. All the topics covered during the workshop are displayed in Table 1.

**Evaluation**

We evaluated the course by analysing the evaluation forms filled by the participants. Each day participants responded anonymously to questions concerning the quality and content of the presentations, and how much they had learnt. On the last day they did an overall assessment of the workshop. Analysis of the evaluation forms shows that the participants were impressed with the quality and content of the presentations and the practical sessions. They appreciated the convivial atmosphere, but some activities were disturbed by poor internet connection (Table 2).

**Outcomes**

At the end of the workshop participants identified topics in which they would like to carry out systematic reviews. Some of these were used as practical examples to determine if reviews had already been done. For the other topics, we hope to assist the participants in determining if reviews exist and the way forward if no reviews currently exist. Some participants have already identified Cochrane Review Groups of interest to them and are in the process of registering titles for systematic reviews. The diversity of research interests and opinions was an enriching experience for both facilitators and participants.

## Conclusion

Participants were impressed with the content and facilitation of this workshop on systematic reviews and meta-analysis. The workshop was an ideal forum for identifying potential authors of systematic reviews and topics of interest for review. The participants were more likely to suggest topics of local relevance. The participants recommended that further support be provided for participants interested in conducting systematic reviews, and that more training should be provided on basic epidemiology, research methods and statistics. We recommend training more participants with a public health background to increase the scope of proposals developed. Facilitators of future systematic review courses should address challenges related to internet access, andragogy and realistic expectations from the participants.

## Acknowledgements 

The workshop was partially funded by The Cochrane Collaboration, The South African Medical Research Council, The Yaoundé Central Hospital, and The Global Health Research Initiative. We would like to thank Taryn Young and Susan Munabi Babigumira for their contributions to this workshop.

## Figures and Tables

**Table 1:** Material covered during fourday systematic review workshop in Cameroon

**Day 1 **
Course objectives
Basic concepts in epidemiology and statistics
What’s so special about systematic reviews?
How to ask answerable questions
Searching and selection of studies
**Day 2**
Assessing risk of bias
Data extraction
The Cochrane Collaboration/ The Cochrane Library
**Day 3**
Data analysis and synthesis
Assessing and interpreting systematic reviews
Practical workshop on searching for reviews
**Day 4**
Overview of health systems and organisation of care
Systematic reviews on health systems and organisation of care
Group work on review questions
Presentation of existing review groups, finalizing a review, publishing tips and useful hints
General discussion on challenges and issues of concern

**Table 2:** Overall assessment of four day systematic review workshop in Cameroon

**Outcomes**	**Satisfactory**	**Good**	**Excellent**
Quality of the lectures		5	7
Quality of the practical exercises		6	6
Quality of the reading material		6	6
Pace of the course	1	7	4
Amount of subject material covered		9	3
